# Supplementary material for: Patient preferences and cost-benefit of hypertension and hyperlipidemia collaborative management model between pharmacies and primary care in Portugal: A discrete choice experiment alongside a trial (USFarmácia®)
Source: PLoS One. 2023 Oct 5;18(10):e0292308. doi: 10.1371/journal.pone.0292308 (PMC10553278; doi:10.1371/journal.pone.0292308)
Supplement: S1 Appendix — (PDF) [file pone.0292308.s001.pdf]

S1 Appendix. Questionnaire on Patient Preferences (version Q1)

Research Study
Hypertension and/or Hyperlipidemia

Telephone Survey – QUESTIONNAIRE ON PATIENT PREFERENCES

1) Good morning (good evening).

(2a) May I speak to Mr./Ms. [NAME]?

[PAUSE– IF PATIENT, GO TO (3).

IF SOMEONE ELSE (NOT PATIENT), GO BACK TO (1), THEN GO TO (2b) AND FINALLY GO TO (3)].

2b) Am I speaking with Mr./Ms. [NAME]?

(3) My name is _____ and, as previously agreed with you, I am calling on behalf of the [INSTITUTION] for the last interview. In this final interview, we would like to know your opinion and preferences on 4 scenarios of different pharmacy intervention models for patients with high blood pressure and/or high blood cholesterol.

There are no right or wrong answers, we just want to know your opinion.

I remind you will not be identified; your answers are confidential and will be used for statistical purposes only. This interview will last approx. 15 min. May we start?

I now ask you to please have your ID Card or any other document containing your NHS number ready.

IN DATABASE

ID1. ID Questionnaire: **Q1**

ID2. NHS Number *(carried forward from trial survey T12)*: |___|___|___|___|___|___|___|___|___|

ID3. Interviewer code: |___|___|

TO FILL IN BY INTERVIEWER

R1. Date(s) of tentative calls prior to this date (if existing):

Tentative	Date and time of call		Tentative	Date and time of call	
1st	___/___/ 2020	___:___	5th	___/___/ 2020	___:___
2nd	___/___/ 2020	___:___	6th	___/___/ 2020	___:___
3rd	___/___/ 2020	___:___	7th	___/___/ 2020	___:___
4th	___/___/ 2020	___:___	8th	___/___/ 2020	___:___

R2. Date of this interview: ___/___/2020 **R3. Start time**: _____ : _____

- **P01.** [VALIDATE PATIENT'S NAME] __ *OK, validated.*
- **P02.** [VALIDATE NHS NUMBER] – Could you please check your ID Card and tell me your NHS Number? It is on the back, last number on your right: __ *OK, validated*.
  If patient provides different number from database *(ask to confirm)*: ____________________

Copyright © 2020 _Q1 – HTA-Disl FINAL PREF_VF_20200130_ENGLISH VERSION (TRANSLATED FROM ORIGINAL IN PORTUGUESE)

S1 Appendix. Questionnaire on Patient Preferences (version Q1)

Please imagine the following scenario.

Your blood pressure and/or cholesterol values have been above normal for some time OR you have been experiencing discomfort with your medication for high blood pressure or cholesterol.

Now imagine there are 2 types of pharmacy services that differ from one another in 5 dimensions:

1) **Model** of pharmacy intervention.

2) **Integration** with primary care.

3) **Waiting time** to get requested medical appointment.

4) **Chance** of having a stroke in the next 5 years.

5) **Annual cost** to the National Health Service (NHS).

The scenario has 2 options, and you need to choose one – service A or service B.

P1. SCENARIO 1.13

[PLEASE READ FIRST ALL ATTRIBUTES OF SERVICE A AND THEN ALL ATTRIBUTES OF SERVICE B!!]

For service A… [FOR EACH ROW: READ TEXT IN ATTRIBUTE + TEXT IN SERVICE A].

For service B… [FOR EACH ROW: READ TEXT IN ATTRIBUTE + TEXT IN SERVICE B].

Which services A or B would you choose? [REPEAT READING IF NECESSARY].

	ATTRIBUTE	SERVICE A	SERVICE B
1	The model of Pharmacy intervention comprises:	5-minute at counter for a basic medication check of your prescription medicines.	15-minute in private office every 3 months for BP measurement and medication review of your medicines for high blood pressure and/or cholesterol.
2	The degree of integration with primary care is:	Weak – NO protocols pre-agreed with physicians and it is not possible to schedule medical appointment with your GP via pharmacy IT system	Full – protocols pre-agreed with physicians AND it is possible to schedule medical appointment with your GP via pharmacy IT system
3	The waiting time to get requested medical appointment is:	48 hrs (urgent) / 30 days (not urgent)	7 days (urgent) / 45 days (not urgent)
4	The chance of having a stroke in the next 5 years:	Is much lower	Is slightly lower
5	The annual cost to the NHS is:	76,00€	51,00€
	WHICH SERVICES A OR B WOULD YOU CHOOSE?	☐	☐

Forget now this scenario and let us move on to the second scenario.

Again, I will read attributes for service A first and then attributes for service B. When I finish, you will need to choose one – service A or service B.

Copyright © 2020 _Q1 – HTA-Disl FINAL PREF_VF_20200130_ENGLISH VERSION (TRANSLATED FROM ORIGINAL IN PORTUGUESE)

S1 Appendix. Questionnaire on Patient Preferences (version Q1)

P2. SCENARIO 1.19

[PLEASE READ FIRST ALL ATTRIBUTES OF SERVICE A AND THEN ALL ATTRIBUTES OF SERVICE B!!]

For service A… [FOR EACH ROW: READ TEXT IN ATTRIBUTE + TEXT IN SERVICE A].

For service B… [FOR EACH ROW: READ TEXT IN ATTRIBUTE + TEXT IN SERVICE B].

Which services A or B would you choose? [REPEAT READING IF NECESSARY].

	**ATTRIBUTE**	**SERVICE A**	**SERVICE B**
**1**	The model of Pharmacy intervention comprises:	30-minute in private office every 6 months for BP, total cholesterol, LDL, HDL and triglycerides measurement and medication review of ALL your medicines	15-minute in private office every 3 months for BP measurement and medication review of your medicines for high blood pressure and/or cholesterol.
**2**	The degree of integration with primary care is:	Weak – NO protocols pre-agreed with physicians and it is not possible to schedule medical appointment with your GP via pharmacy IT system	Weak – NO protocols pre-agreed with physicians and it is not possible to schedule medical appointment with your GP via pharmacy IT system
**3**	The waiting time to get requested medical appointment is:	Same day (urgent) / 15 days (not urgent)	Same day (urgent) / 15 days (not urgent)
**4**	The chance of having a stroke in the next 5 years:	Is much lower	Is the same
**5**	The annual cost to the NHS is:	0€	0€
	WHICH SERVICES A OR B WOULD YOU CHOOSE?	☐	☐

Forget now this scenario and let us move on to the third scenario.

Copyright © 2020 _Q1 – HTA-DisI FINAL PREF_VF_20200130_ENGLISH VERSION (TRANSLATED FROM ORIGINAL IN PORTUGUESE)

S1 Appendix. Questionnaire on Patient Preferences (version Q1)

P3. SCENARIO 1.25

[PLEASE READ FIRST ALL ATTRIBUTES OF SERVICE A AND THEN ALL ATTRIBUTES OF SERVICE B!!]

For service A… [FOR EACH ROW: READ TEXT IN ATTRIBUTE + TEXT IN SERVICE A].

For service B… [FOR EACH ROW: READ TEXT IN ATTRIBUTE + TEXT IN SERVICE B].

Which services A or B would you choose? [REPEAT READING IF NECESSARY].

	ATTRIBUTE	SERVICE A	SERVICE B
**1**	The model of Pharmacy intervention comprises:	15-minute in private office every 3 months for BP measurement and medication review of your medicines for high blood pressure and/or cholesterol	15-minute in private office every 3 months for BP measurement and medication review of your medicines for high blood pressure and/or cholesterol
**2**	The degree of integration with primary care is:	Full – protocols pre-agreed with physicians AND it is possible to schedule medical appointment with your GP via pharmacy IT system	Partial – protocols pre-agreed with physicians BUT it is not possible to schedule medical appointment with your GP via pharmacy IT system
**3**	The waiting time to get requested medical appointment is:	7 days (urgent) / 45 days (not urgent)	Same day (urgent) / 15 days (not urgent)
**4**	The chance of having a stroke in the next 5 years:	Is slightly lower	Is much lower
**5**	The annual cost to the NHS is:	51,00€	76,00€
	WHICH SERVICES A OR B WOULD YOU CHOOSE?	☐	☐

Forget now all these scenarios and let us move on to the last scenario.

Copyright © 2020 _Q1 – HTA-Disl FINAL PREF_VF_20200130_ENGLISH VERSION (TRANSLATED FROM ORIGINAL IN PORTUGUESE)

S1 Appendix. Questionnaire on Patient Preferences (version Q1)

P4. SCENÁRIO 1.36

[PLEASE READ FIRST ALL ATTRIBUTES OF SERVICE A AND THEN ALL ATTRIBUTES OF SERVICE B!!]

For service A… [FOR EACH ROW: READ TEXT IN ATTRIBUTE + TEXT IN SERVICE A].

For service B… [FOR EACH ROW: READ TEXT IN ATTRIBUTE + TEXT IN SERVICE B].

Which services A or B would you choose? [REPEAT READING IF NECESSARY].

	ATTRIBUTE	SERVICE A	SERVICE B
1	The model of Pharmacy intervention comprises:	15-minute in private office every 3 months for BP measurement and medication review of your medicines for high blood pressure and/or cholesterol	5-minute at counter for a basic medication check of your prescription medicines
2	The degree of integration with primary care is:	Full – protocols pre-agreed with physicians AND it is possible to schedule medical appointment with your GP via pharmacy IT system	Partial – protocols pre-agreed with physicians BUT it is not possible to schedule medical appointment with your GP via pharmacy IT system
3	The waiting time to get requested medical appointment is:	Same day (urgent) / 15 days (not urgent)	7 days (urgent) / 45 days (not urgent)
4	The chance of having a stroke in the next 5 years:	Is the same	Is the same
5	The annual cost to the NHS is:	30,00€	51,00€
	WHICH SERVICES A OR B WOULD YOU CHOOSE?	□	□

We are almost done. I have just 5 quick questions for statistics. I remind you that your answers will be kept anonymous and confidential.

P5. Do you have a General Practitioner (GP) assigned to you?

__ Yes __ No __ I do not know

P6. Do you pay NHS user charges?

__ Yes __ No __ I do not know

P7. Do you have a health sub-system or health insurance?

__ Yes. Which one _______ __ No __ I do not know

P8. [FOR WOMEN UNDER 45 OR BIRTH DATE AFTER 1974]: **Are you / were you pregnant sometime between June 2016 and now?**

__ Yes __ No

Copyright © 2020 _Q1 – HTA-DisI FINAL PREF_VF_20200130_ENGLISH VERSION (TRANSLATED FROM ORIGINAL IN PORTUGUESE)

P9. Last question – how did you find this interview? (You may choose more than one of the following options):

__ Interesting

__ Too long

__ Hard to understand

__ Not realistic

__ Other (PLEASE EXPLAIN) ___________________

Thank you. This was the last telephone interview. On behalf of the [INSTITUTION], we thank you for your time.

R4. End time: _____ : ____

TO BE ANSWERED BY THE INTERVIEWER

R5. In your opinion, was this interview difficult for this patient:

__ Not too difficult

__ Somewhat difficult

__ Very difficult

R6. Do you have any other comments?
