# Supplementary material for: Patient preferences and cost-benefit of hypertension and hyperlipidemia collaborative management model between pharmacies and primary care in Portugal: A discrete choice experiment alongside a trial (USFarmácia®)
Source: PLoS One. 2023 Oct 5;18(10):e0292308. doi: 10.1371/journal.pone.0292308 (PMC10553278; doi:10.1371/journal.pone.0292308)
Supplement: Attachment — Submitted filename: PONE-D-22-23923.docx [file pone.0292308.s002.docx]

Patient preferences and cost-benefit of hypertension and hyperlipidemia collaborative management model between pharmacies and primary care in Portugal: A discrete choice experiment alongside a trial (USFarmácia®)

Thanks for the opportunity to review your article, Patient preferences and cost-benefit of hypertension and hyperlipidemia collaborative management model between pharmacies and primary care in Portugal: A discrete choice experiment alongside a trial (USFarmácia®). This is a well written article and I have minor comments for clarification:

Further explanation about the difference between the WTP/WTA model in DCEs should be clarified (and specific wording used for this experiment) in light also of the literature <https://doi.org/10.1111/j.1524-4733.2008.00340.x>.

In the discussion the authors mentioned that sample size calculations were not provided as they had to rely on a predefined number of patients enrolled in the trial. Still, it would be important to understand whether the sample size available was suitable for the DCE exercise (and calculations should be reported in the methods section).

The choice of the regression model is not clarified. I think the readers would appreciate to know whether alternative models have been tested (and the selection criteria applied to find the model with the best fit).
