# Supplementary material for: Patient preferences and cost-benefit of hypertension and hyperlipidemia collaborative management model between pharmacies and primary care in Portugal: A discrete choice experiment alongside a trial (USFarmácia®)
Source: PLoS One. 2023 Oct 5;18(10):e0292308. doi: 10.1371/journal.pone.0292308 (PMC10553278; doi:10.1371/journal.pone.0292308)
Supplement: Attachment — Submitted filename: DCE Review.docx [file pone.0292308.s003.docx]

Thank you for the opportunity to review the manuscript “Patient preferences and cost-benefit of hypertension and 1 hyperlipidemia collaborative management model between pharmacies and primary care in Portugal: A discrete choice experiment alongside a trial (USFarmácia®).” This is a challenging DCE study which looks to identify patient preferences for a collaborative medication management model. I believe using these methods to inform intervention design is important and has the potential to inform patient-centered services alongside qualitative research and patient-public involvement. I have several concerns related to the DCE methodology used, most notably limitations from attributes, the potential implications of cognitive burden, the small sample size, and how efficiency/blocking/choice tasks were calculated and determined.

**Abstract**

Methods: please provide additional methodology necessary to understand how the results for each objective were intended to be collected and analyzed. What was done for the economic evaluation?

Additionally, where was the convenience sample from?

Methods should also included attribute levels if attribute levels are to be discussed in the abstract results.

Results: How many individuals responded to the survey?

The results regarding the economic model and the acceptable monetary amount for preferred service is confusing. Was this calculated using the cost attribute or by some other means?

**Main Text**

Introduction: overall the introduction is fragmented and difficult to appreciate the relevance of certain paragraphs. For example, the second paragraph on DCE use for public policy. This seems to separate two more related paragraphs. Please revise for clarity and continuity.

The use of WTP and integration into a CBA is an interesting and novel approach.

The review of pharmacy DCE literature is dated, as several studies have recently been published:

<https://www.sciencedirect.com/science/article/abs/pii/S0168851021002207>

<https://www.sciencedirect.com/science/article/abs/pii/S1551741123000281>

<https://www.sciencedirect.com/science/article/abs/pii/S1551741118303061>

<https://www.sciencedirect.com/science/article/pii/S0738399120306698>

<https://pubmed.ncbi.nlm.nih.gov/33608244/>

Consider either consolidating the review to emphasize why existing articles are relevant to this study or expand the review to included more recent articles.

Methods

Page 4 Line 87-88: what are the inclusion criteria?

The methods for attribute selection are rigorous and exceptional, using both qualitative and quantitative data. Importantly, how were data from the focus group used to inform attribute selection? Was qualitative analysis performed to identify attributes?

While I appreciate the justification for how attribute levels were selected, the way attribute levels are introduced are a bit confusing (e.g. “best” level 3). Please clarify what each of these terms mean and what the levels of the attributes are corresponding to each of the terms.

Box 1, unsure if this is a customary data presentation technique in the journal but this type of information is more commonly displayed in table format.

When analyzing orthogonal design efficiency, did the R package use consider the 9 blocks and 4 choice sets/respondent. Other software (Sawtooth) which is used for D-optimal orthogonal design generation incorporates these changes into the design efficiency calculation. Not including blocking and respondent choice set exposure has the potential to decrease design efficiency if not included in the efficiency calculation.

Provide justification for why an opt-out option was not included.

Were any methods used to assess internal validity and reliability outside of pretesting? Typically in DCE studies, a dominant choice will be used to test the theoretical validity of the choice tasks, suggesting that an obviously better choice will be selected more often.

The survey in appendix 1 was very helpful for understanding the assessment instrument.

**Results**

Were the 122 respondents included in the calculation for efficiency? Most DCE studies require considerably larger samples to assure accuracy of the findings. Most studies in pharmacy have aimed for 500. Is there justification for why a sample of 122 would be considered sufficient related to the efficiency, blocking, and items? This is only 13 responses/block which is concerning.

Page 9 and 10, Lines 240-242, I see that there is mention of an alternative profile with the best level of one attribute always in all 4 choice sets. Is this the internal consistency/validity check I mentioned earlier? If so please include earlier and describe in more detail. Please clarify what this alternative choice task was designed to assess and following this, what was the justification for including them in the analysis.

Typically in DCE studies, we would see attribute importance values (AIV) as a means to understand the relative value of each attribute. It seems WTA provides somewhat similar information, but AIV would be important to include.

**Discussion**

The discussion requires considerable revision to place the findings in the context of existing literature, with more thorough description of references studies and how these results relate.

Page 16 Line 307: Given that the chance of stroke did not contribute to decision-making and patients may not have understood the attribute, it makes the reader question the cognitive burden placed on respondents. Looking at the survey items, and appreciating that individuals were being read the choices, it is possible that individuals could not recall and/or process all variables due to the length of the attribute level descriptions. Were these concerns addressed and evaluated during DCE survey pilot?

**Limitations**

I do not agree that DCE works best with face-to-face interviews. Paper or electronic surveys are also very efficient ways to have individuals complete DCE studies and allow individuals to review attributes in each choice task.

A major limitation is that the sample is small and DCE methodology are potentially inappropriate, a consideration which cannot be justified by constraints from available trial patients.

Having an attribute which performs poorly also has the potential to bias DCE results considerably. If respondents are not considering all attributes equally or have difficulty interpreting or understanding an attribute, they are likely to assign additional value to other variables. Thee attribute performing poorly is concerning for the overall study results.
