# Supplementary material for: Patient preferences and cost-benefit of hypertension and hyperlipidemia collaborative management model between pharmacies and primary care in Portugal: A discrete choice experiment alongside a trial (USFarmácia®)
Source: PLoS One. 2023 Oct 5;18(10):e0292308. doi: 10.1371/journal.pone.0292308 (PMC10553278; doi:10.1371/journal.pone.0292308)
Supplement: Attachment — Submitted filename: Response to reviewers.docx [file pone.0292308.s004.docx]

**Patient preferences and cost-benefit of hypertension and hyperlipidemia collaborative management model between pharmacies and primary care in Portugal: A discrete choice experiment alongside a trial (USFarmácia®)**

**Reviewer 1**

*Thank you for your review of our manuscript. We appreciated your questions as they provided us with an opportunity to (hopefully) improve this manuscript. We have answered each comment below. Changes and/or new text in the manuscript are highlighted in YELLOW.*

| **#** | **Comment** | **Reply** |
| --- | --- | --- |
| **1** | Further explanation about the difference between the WTP/WTA model in DCEs should be clarified (and specific wording used for this experiment) in light also of the literature <https://doi.org/10.1111/j.1524-4733.2008.00340.x>. | *Thank you for calling our attention to this. You are right, this was not clear. We added this explanation under Methods (Annual cost to the NHS) as follows:*  The value can be studied from the perspective of WTP (costs defined as extra payment), the maximum amount a person would be willing to offer for a good, or of WTA (costs defined as discount or compensation), the minimum monetary amount required for an individual to forgo some good, or to bear some harm [47,48]. WTA measures the amount of money that is required *after* the change to make a respondent’s level of utility the same as before the change [48]. |
| **2** | In the discussion the authors mentioned that sample size calculations were not provided as they had to rely on a predefined number of patients enrolled in the trial. Still, it would be important to understand whether the sample size available was suitable for the DCE exercise (and calculations should be reported in the methods section). | *We added a paragraph under Methods (Data Collection, DCE Survey), as requested, as follows:*  We performed the sample size calculation for our 6-month effectiveness trial resulting in a required minimum sample size of 322 hypertensive patients, considering a 20% drop-out rate, 80% power, and a 5% significance level to detect a change in our desired outcome. We also estimated the required minimum sample size for our DCE.  For sample size calculation, we took note of the “rule of thumb” method of Johnson and Orme for DCEs [54,55] which suggests that the sample size required for the main effects depends on the number of choice tasks (t), the number of alternatives (a), and the number of analysis cells (c) according to the following equation:  N > 500c / (t x a)  Considering the main effects, we would require a minimum of 250 patients. However, we had to use available 122 trial patients who were recruited from trial patients who had replied to the 12-month telephone survey and reconfirmed consent.  *We also adjusted the sentence under Discussion (Limitations) as follows:*  We could not reach the minimum sample size required according to Johnson and Orme for DCEs [54,55]. We acknowledge the sample is small, but we did plan for an adequate sample size. The sample size estimate for the effectiveness trial would be more than sufficient for our trial exit DCE survey. Recruitment efforts aimed, therefore, at achieving 322 hypertensive patients. However, this was a pragmatic controlled trial to assess effectiveness under real-world conditions where not all variables can be controlled by the researcher despite the planning. And, in practice, the research team faced unexpected external challenges which affected recruitment and prevented it from reaching the desired sample size. |
| **3** | The choice of the regression model is not clarified. I think the readers would appreciate to know whether alternative models have been tested (and the selection criteria applied to find the model with the best fit). | *Thank you very much for this comment. We agree with the reviewer and we added a paragraph under Methods (Data analysis) clarifying this, as requested:*  The primary choice for this model assumed intervention trial patients shared many baseline characteristics since they all had to meet pre-defined inclusion criteria, and that they had experienced the same standardized patient care collaborative intervention, thereby reducing the risk of preference heterogeneity and variance across choice tasks [41]. In addition, as this was a trial exit interview, we had to work with available patients and did not have a large enough sample size to test alternative models.  *We further reported this as a limitation under Discussion (Limitations):*  We did not test alternative regression models, such as Random-Parameters Logit (RPL), Hierarchical Bayes, or Latent Class Models (LCM) to allow for unobserved heterogeneity and compare the consistency of results, as we did not have a large enough sample size [41]. |

**Reviewer 2**

*Thank you for your through review of our manuscript. We appreciate the time you took, your questions, and careful remarks and suggestions. We have answered each comment below. Changes and/or new text in the manuscript are highlighted in YELLOW.*

*We hope the revised version addresses your concerns and we thank you for assisting us in presenting an improved manuscript.*

| **#** | **Comment** | **Reply** |
| --- | --- | --- |
|  | **Abstract** |  |
| **1** | Methods: please provide additional methodology necessary to understand how the results for each objective were intended to be collected and analyzed. What was done for the economic evaluation? | *We adjusted the sentence in the Abstract (under Methods) to clarify this:*  We performed a DCE telephone interview with a sample of hypertension and hyperlipidemia trial patients 12 months after trial onset.  *We also added a sentence to explain the economic evaluation method:*  We subtracted the estimated annual incremental trial costs from the mean WTA (Net Benefit) for CBA. |
| **2** | Additionally, where was the convenience sample from? | *We adjusted the sentence in the Abstract (under Methods) to clarify this:*  We performed a DCE telephone interview with a sample of hypertension and hyperlipidemia trial patients 12 months after trial onset. |
| **3** | Methods should also included attribute levels if attribute levels are to be discussed in the abstract results. | *We detailed the sentence in the Abstract (under Methods) as requested:*  We used five attributes (levels): waiting time to get urgent/not urgent medical appointment (7 days/45 days; 48 hrs./30 days; same day/15 days), model of pharmacy intervention (5-min. counter basic check; 15-min. office every 3 months for BP and medication review of selected medicines; 30-min. office every 6 months for comprehensive measurements and medication review of all medicines), integration with primary care (weak; partial; full), chance of having a stroke in 5 years (same; slightly lower; much lower), and annual cost to the NHS (0€; 30€; 51€; 76€). |
| **4** | Results: How many individuals responded to the survey? | *We added a sentence in the Abstract (under Results) as follows:*  A total of 122 patients completed the survey. |
| **5** | The results regarding the economic model and the acceptable monetary amount for preferred service is confusing. Was this calculated using the cost attribute or by some other means? | *First, we calculate WTA annual cost to the NHS (ratio of the regression coefficient for each attribute to the cost coefficient). The WTA is reported in Table 2.*  *Next, for the intervention patients only, we take the WTA for the preferred attribute level. This is reported in Table 3 and is the marginal WTA (versus the reference value which is the least preferred scenario). We then sum all WTA for the three attributes (WTA for the preferred trial-like scenario) that are significant (877.00€).*  *For the CBA, we used Net Benefit (WTA for the preferred (trial-like) scenario – Trial Costs). Trial incremental costs are published elsewhere in the cost-effectiveness study of this trial.*  *The Net Benefit is the difference between the intervention patients WTA minus the actual incremental trial costs – the monetary value of intervention patients’ welfare surplus for the preferred care model.* |
|  | **Main text** |  |
| **6** | Introduction: overall the introduction is fragmented and difficult to appreciate the relevance of certain paragraphs. For example, the second paragraph on DCE use for public policy. This seems to separate two more related paragraphs. Please revise for clarity and continuity. | *Thank you very much for this comment. We agree with the reviewer, and we sought to improve this by reordering the paragraphs in the introduction.*  *In short, we start with the DCE, WTP/WTA, and CBA concepts. Then we present the existing evidence on DCEs used for public policy. Next, the evidence on DCEs for pharmacy services in other countries. Then, we narrow down to the DCE experiences in Portugal. We further explain the relevance of the selected disease areas. We briefly present the trial, and close with the aims of our study.* |
| **7** | The review of pharmacy DCE literature is dated, as several studies have recently been published:  <https://www.sciencedirect.com/science/article/abs/pii/S0168851021002207>  <https://www.sciencedirect.com/science/article/abs/pii/S1551741123000281>  <https://www.sciencedirect.com/science/article/abs/pii/S1551741118303061>  <https://www.sciencedirect.com/science/article/pii/S0738399120306698>  <https://pubmed.ncbi.nlm.nih.gov/33608244/>  Consider either consolidating the review to emphasize why existing articles are relevant to this study or expand the review to included more recent articles. | *We thank the reviewer for this comment. However, we would like to clarify that the review of pharmacy DCE literature presented is restricted to patient preferences for a community pharmacy-delivered patient care service. It does not include preferences not related to a patient care service(s) or provided by pharmacists in other settings outside community pharmacy. We agree this may not be clear and we sought to improve this in the text.*  *In addition, since we submitted this manuscript to this journal (Aug 2022), only one DCE study meeting our criteria seems to have been published, by Murry LT and colleagues, from USA, in May 2023 (your second reference) on medication therapy management, according to our new search for the period 2021-2022. Thus, we added it to the review of pharmacy literature reported in Introduction (reference 30).*  Since then, 12 more DCE studies were published on patient preferences for a pharmacy-delivered patient care service: : 2 on minor ailments [25,26], 4 on medication therapy management or disease management [27-30],… |
|  | **Methods** |  |
| **8** | Page 4 Line 87-88: what are the inclusion criteria? | *Thank you for noting this. We added a sentence under Methods (Trial-Based Study) as follows:*  Patients were recruited in pharmacies according to inclusion criteria: adult NHS patients on medication for hypertension and/or hyperlipidemia, either new to therapy or usual medication users, preferably with baseline blood pressure and total cholesterol above reference values. Intervention patients were also mobile phone users (to receive SMS refill reminders), consenting for data exchange between pharmacists and general practitioners, and holders of a patient record |
| **9** | The methods for attribute selection are rigorous and exceptional, using both qualitative and quantitative data. Importantly, how were data from the focus group used to inform attribute selection? Was qualitative analysis performed to identify attributes? | *Thank you. We performed a trial exit patient focus group using a purposive quota sampling of trial intervention patients who experienced some collaborative features between pharmacies and primary care. This is a separate research study under submission. We aimed to explore patient-reported experiences and expectations about their disease condition and collaborative attributes and levels between pharmacies and primary care. The focus was conducted face-to-face, audio-recorded, transcribed, and thematically analyzed. The content thematic analysis contributed, with other sources, to identify DCE attributes and levels.*  *We added a brief clarification under Methods (DCE Attributes and Levels):*  2) a trial exit patient focus group using thematic analysis; |
| **10** | While I appreciate the justification for how attribute levels were selected, the way attribute levels are introduced are a bit confusing (e.g. “best” level 3). Please clarify what each of these terms mean and what the levels of the attributes are corresponding to each of the terms. | *We adjusted the sentence under Methods (DCE Attributes and Levels):*  Levels were informed by the different sources as outlined further. Level 1 was defined as usual care in all attributes (almost no collaborative or integrated care). The remaining levels sought to illustrate possible responses within a collaborative care model. Level 2 represents improved care (vs usual care) and level 3 represents the care level provided in the intervention arm of the collaborative care trial (the highest form of collaborative care) for all attributes except cost. Levels of the cost attribute are explained further.  *We tried to clarify this further under each attribute description:*  **Model of pharmacy intervention**  …Level 2 is a more frequent, yet less comprehensive intervention. Level 3 is a less frequent yet more comprehensive model of pharmacy intervention (the highest form of pharmacy intervention).  **Integration with primary care**  We selected usual care which is almost no integration (level 1) and two other options (levels 2 and 3) describing the degree of integration with primary care as planned for the trial intervention arm expressed in protocols with physicians and scheduling medical appointments directly from the pharmacy software. In level 2 there is partial integration. Level 3 corresponds to the full integration with primary care featured in the intervention arm of the collaborative trial.  **Waiting time to get requested medical appointment**  We defined level 3 as same day (urgent)/15 days (not urgent) and “intermediate” based on the Maximum Waiting Time for referrals to primary care defined in legislation [46]. This would be the “best” level for waiting time to pursue in future collaborative care. Level 2 was defined as 48 hrs. (urgent)/ 30 days (not urgent) options as a first step towards “best” adopted for the trial intervention arm. …We defined possible usual care (level 1) as 7 days (urgent)/45 days (not urgent)..  **Chance of having a stroke in 5 years**  … Level 1 corresponds to usual care (no change). In level 2, the chance of stroke is slightly lower as a result of some collaborative care. In level 3, the chance of stroke is much lower as a consequence of the desired highest level of collaborative care, |
| **11** | Box 1, unsure if this is a customary data presentation technique in the journal but this type of information is more commonly displayed in table format. | *We labelled it “Box” to differentiate from Tables which usually appear in Results section and depict study data. This has been used in other journals, but we can change if the editors of PLoS One prefer it otherwise.* |
| **12** | When analyzing orthogonal design efficiency, did the R package use consider the 9 blocks and 4 choice sets/respondent. Other software (Sawtooth) which is used for D-optimal orthogonal design generation incorporates these changes into the design efficiency calculation. Not including blocking and respondent choice set exposure has the potential to decrease design efficiency if not included in the efficiency calculation. | *First, we performed the fractional factorial design in R which resulted in 36 orthogonal combinations. Then because we could not have patients respond to 36 choice sets over the phone, we discussed within the research team on potential feasible combinations: either 18x2, 12x3, 9x4, 6x6. The latter would increase the burden for patients, being a telephone interview with no visualization. Therefore, we opted for 9x4. The allocation of combination sets per each block was, however, performed by R to achieve the maximum reported efficiency.* |
| **13** | Provide justification for why an opt-out option was not included. | *We sought to improve the justification under Discussion (Limitations):*  We did not include an opt-out alternative, (e.g. patients’ current pharmacy service features), but we did not have a large enough sample size to account for the loss of preference data. Choosing an opt-out alternative does indicate the relative preference of patients who do not wish to choose between available alternatives, but an opt-out alternative may have implications as patients need to be censored in preference assessment. Hence, a large enough sample size is required for assessing preferences on attributes and levels to account for the loss of patients who choose an opt-out alternative. |
| **14** | Were any methods used to assess internal validity and reliability outside of pretesting? Typically in DCE studies, a dominant choice will be used to test the theoretical validity of the choice tasks, suggesting that an obviously better choice will be selected more often. | *We answered to this question further down below under your question #16 including changes made in the manuscript, as requested.* |
|  | **Results** |  |
| **15** | Were the 122 respondents included in the calculation for efficiency? Most DCE studies require considerably larger samples to assure accuracy of the findings. Most studies in pharmacy have aimed for 500. Is there justification for why a sample of 122 would be considered sufficient related to the efficiency, blocking, and items? This is only 13 responses/block which is concerning. | *The sample size is not a parameter in the calculation of design efficiency performed in R. The relative efficiency was about achieving the orthogonal design for the pairwise of profile alternatives considering the most efficient combinations of attributes and levels regardless of sample size which is not a parameter.*  *However, we do acknowledge the low sample size, but we did plan for the adequate sample size.*  *We added a detailed explanation about the sample size issue under Discussion (Limitations) which we report in reply to your question #21.* |
| **16** | Page 9 and 10, Lines 240-242, I see that there is mention of an alternative profile with the best level of one attribute always in all 4 choice sets. Is this the internal consistency/validity check I mentioned earlier? If so please include earlier and describe in more detail. Please clarify what this alternative choice task was designed to assess and following this, what was the justification for including them in the analysis. | *Yes. We detailed this under Methods (Data analysis) as requested:*  We assessed the quality of responses by evaluating the internal validity of the data and looking for respondents who always chose the alternative profile with the best level of one attribute in all 4 choice sets because preferences that are dominated by a single attribute can bias model estimation.  *We reported the results of this internal validity under Results with added detail:*  Between 0 and 14% of respondents chose the alternative profile with the best level of one attribute always in all 4 choice sets: 9 (7%) patients in model of intervention; 5 (4%) patients in integration with primary care; 17 (14%) patients in waiting time to get requested medical appointment; and 3 (2%) patients in annual cost to the NHS. Since these are few patients and preferences do not seem to be dominated by a single attribute, we included these patients in the analysis. |
| **17** | Typically in DCE studies, we would see attribute importance values (AIV) as a means to understand the relative value of each attribute. It seems WTA provides somewhat similar information, but AIV would be important to include. | *We added a sentence under Methods (Data analysis):*  We calculated the relative importance of each attribute which is the ratio of each attribute’s utility range (difference between the highest and lowest utility value) to the sum of the utility range for all attributes.  *And we reported calculations under Results:*  Since the coefficients of the chance of stroke and the annual cost to the NHS were not statistically significant, we just calculated the relative importance of the remaining attributes. Again, we confirmed waiting time to get a medical appointment had the highest relative importance (57%) followed by the model of pharmacy intervention (25%) and integration with primary care (18%). |
|  | **Discussion** |  |
| **18** | The discussion requires considerable revision to place the findings in the context of existing literature, with more thorough description of references studies and how these results relate. | *Thank you for this relevant remark. We placed findings in the context of studies conducted in Portugal but not in the context of international literature. Thank you for pointing this out.*  *We added a sentence under Discussion (Summary of Key Findings) as follows:*  Our results are consistent with international DCE patient enhanced care pharmacy studies where patients express a preference for a shorter waiting time [15,16,18,25,26,28,33,34,36], a similar model of pharmacy intervention [15,17,19,20,24,28,31,35,36], and the chance of best treatment [18,28,29]. There are still very few DCE studies published on pharmacy-led integrated or collaborative care with physicians, but these also confirm preferences for integration with physicians [27,28]. Almost all studies framed cost as WTP which makes it difficult to compare but cost is a very strong preference for patients in all these studies when considering an enhanced pharmacy care model or intervention. In addition, the only study which incorporated WTP in a CBA model and in trial patients who experienced an innovative medication-related service also demonstrated a positive Net Benefit [28]. |
| **19** | Page 16 Line 307: Given that the chance of stroke did not contribute to decision-making and patients may not have understood the attribute, it makes the reader question the cognitive burden placed on respondents. Looking at the survey items, and appreciating that individuals were being read the choices, it is possible that individuals could not recall and/or process all variables due to the length of the attribute level descriptions. Were these concerns addressed and evaluated during DCE survey pilot? | *We believe the reason why patients may have not fully understood the chance of stroke attribute is related to the concept of “chance” or probabilities in risk, which has also been reported in the literature. We had stated this under Discussion (Limitations):*  The survey version used in the pretest included probabilities in the risk reduction attribute and this was not well understood which has been reported in previous research [61]. Despite the attempt to improve the final version, it probably remained an issue.  *We do agree, however, it is possible that patients could not recall and/or process all variables (but not necessarily just the chance of stroke attribute. Therefore, we adjusted the text under Discussion (Limitations):*  We used a telephone survey as this was used to collect other patient-reported trial data. We tried to minimize patient burden and risk of poor recall of all variables in a telephone interview with no visualization by 1) keeping choice sets to four; 2) requesting patients to keep a pen and paper handy following recommendations from the pretest; 3) using CATI technology and trained interviewers; 4) providing detailed instruction to the interviewer in the survey before each scenario to standardize the approach and to allow for repetition before collecting the patient’s response. |
|  | **Limitations** |  |
| **20** | I do not agree that DCE works best with face-to-face interviews. Paper or electronic surveys are also very efficient ways to have individuals complete DCE studies and allow individuals to review attributes in each choice task. | *Yes, we fully agree with you and adjusted the sentence to make it clearer:*  DCE is a complex method that works best through face-to-face interviews in older or less literate populations.  *Yet, we had acknowledged other possibilities for this population under Discussion (Limitations):*  The self-administered online survey was not feasible due to the age and literacy level of our population. It could be interesting to explore online surveys in older populations through SMS or WhatsApp® in the future. |
| **21** | A major limitation is that the sample is small and DCE methodology are potentially inappropriate, a consideration which cannot be justified by constraints from available trial patients. | *We acknowledge this limitation and we sought to provide more detailed explanation under Methods:*  We performed the sample size calculation for our 6-month effectiveness trial resulting in a required minimum sample size of 322 hypertensive patients, considering a 20% drop-out rate, 80% power, and a 5% significance level to detect a change in our desired outcome. We also estimated the required minimum sample size for our DCE.  For sample size calculation, we took note of the “rule of thumb” method of Johnson and Orme for DCEs [54,55] which suggests that the sample size required for the main effects depends on the number of choice tasks (t), the number of alternatives (a), and the number of analysis cells (c) according to the following equation:  N > 500c / (t x a)  Considering the main effects, we would require a minimum of 250 patients. However, we had to use available 122 trial patients who were recruited from trial patients who had replied to the 12-month telephone survey and reconfirmed consent.  *And under Discussion (Limitations):*  We could not reach the minimum sample size required according to Johnson and Orme for DCEs [54,55]. We acknowledge the sample is small, but we did plan for an adequate sample size. The sample size estimate for the effectiveness trial would be more than sufficient for our trial exit DCE survey. Recruitment efforts aimed, therefore, at achieving 322 hypertensive patients. However, this was a pragmatic controlled trial to assess effectiveness under real-world conditions where not all variables can be controlled by the researcher despite the planning. And, in practice, the research team faced unexpected external challenges which affected recruitment and prevented it from reaching the desired sample size. |
| **22** | Having an attribute which performs poorly also has the potential to bias DCE results considerably. If respondents are not considering all attributes equally or have difficulty interpreting or understanding an attribute, they are likely to assign additional value to other variables. Thee attribute performing poorly is concerning for the overall study results. | *Yes, we acknowledge, and we added a sentence under Discussion (Limitations) as follows:*  We acknowledge that having an attribute that may have not been fully understood has the potential for patients to assign additional value to other variables which may affect the WTA of each attribute, but we believe it is less likely to affect the relative importance of each attribute or the sum of all attribute WTA used to determine CBA. |
