# Supplementary material for: Patient preferences and cost-benefit of hypertension and hyperlipidemia collaborative management model between pharmacies and primary care in Portugal: A discrete choice experiment alongside a trial (USFarmácia®)
Source: PLoS One. 2023 Oct 5;18(10):e0292308. doi: 10.1371/journal.pone.0292308 (PMC10553278; doi:10.1371/journal.pone.0292308)
Supplement: Attachment — Submitted filename: Response to reviewers_round 2.docx [file pone.0292308.s005.docx]

**Patient preferences and cost-benefit of hypertension and hyperlipidemia collaborative management model between pharmacies and primary care in Portugal: A discrete choice experiment alongside a trial (USFarmácia®)**

**Reviewer 1**

| **#** | **Comment** | **Reply** |
| --- | --- | --- |
| **1** | I reviewed the revised version of the paper and am satisfied with the changes performed to the manuscript apart from one last comment. Page 18, the aggregate willingness to accept monetary amounts in response to policy changes when changing from the least preferred scenario to the most preferred scenario can be calculated in post-estimation welfare analysis using the compensating variation methodology. This should be referenced in the paper. | *Thank you for this additional comment which was missing in the Methods section of this manuscript. We added the following sentence under Methods and respective new reference 62 from Hicks as follows (highlighted in the manuscript in YELLOW):*  We calculated the aggregate WTA annual cost to the NHS for a policy change from a least preferred scenario to the most preferred scenario in post-estimation welfare analysis using the compensating variation methodology [Hicks, JR. The Four Consumer’s Surpluses. The Review of Economic Studies 1943 [cited 2023 Aug 31]; 11(1):31–41. Available from: https://doi.org/10.2307/2967517]. |

**Reviewer 2**

| **#** | **Comment** | **Reply** |
| --- | --- | --- |
|  |  |  |
| **1** | While I still have concerns regarding the sample size and attribute interpretation by participants, I believe the discussion of these limitations is sufficient to warrant publication of this work. Thank you for addressing my initial comments which were extensive. | *Thank you for your thorough review of our manuscript and for assisting us in presenting an improved version.* |
